# Seizure-Inducible Risks of In Vivo Optogenetic Manipulations

**DOI:** 10.3390/biology15181669

**Published:** 2026-09-21

**Authors:** Xutao Zhu, Zhijian Zhang, Yu Tian, Li Wang, Yue Liu, Ronghui Li, Pengjie Wen, Jie Wang, Liping Wang, Qing Liu, Fuqiang Xu

**Affiliations:** 1 Shenzhen Key Laboratory of Viral Vectors for Biomedicine, Shenzhen-Hong Kong Institute of Brain Science, Shenzhen Institutes of Advanced Technology, Chinese Academy of Sciences, Shenzhen 518055, China; 2 State Key Laboratory of Magnetic Resonance and Atomic and Molecular Physics, Wuhan Center for Magnetic Resonance, Innovation Academy for Precision Measurement Science and Technology, Chinese Academy of Sciences, Wuhan 430071, China; zhzhj_hust@126.com (Z.Z.);; 3 Department of Neuroscience and Mahoney Institute for Neurosciences, Perelman School for Medicine, University of Pennsylvania, Philadelphia, PA 19104, USA; 4 Department of Neuroscience, City University of Hong Kong, Kowloon, Hong Kong 999077, China; 5 School of Medicine, Jingchu University of Technology, Jingmen 448000, China; 6 Translational Research Center for the Nervous System, The Brain Cognition and Brain Disease Institute, Shenzhen Institutes of Advanced Technology, Chinese Academy of Sciences, Shenzhen 518055, China; 7 University of Chinese Academy of Sciences, Beijing 100049, China; 8 Center for Excellence in Brain Science and Intelligence Technology, Chinese Academy of Sciences, Shanghai 200031, China

**Keywords:** optogenetics, stimulation parameters, in vivo, seizure-like activities, neuroscience research

## Abstract

Optogenetic manipulation is pivotal in basic neuroscience research and in studying neuropsychiatric disorders, enabling the activation or inhibition of neuronal populations with millisecond precision. However, this technique can induce artificial neuronal hypersynchronization that is rarely observed under physiological conditions. Given that epileptic seizures arise from abnormally synchronized neuronal discharges, the potential for optogenetic stimulation to trigger seizures and confound experimental outcomes warrants close examination. Here, using electrophysiological and behavioral recordings, we demonstrate that even single-trial optogenetic stimulation of CaMKII-positive neurons in the hippocampal CA1 region, anterior piriform cortex (APC), or lateral/medial entorhinal cortex (LEnt or MEnt) can induce seizure-like discharges and behaviors in adult male C57BL/6 mice. Repeated stimulation in the APC, LEnt, or MEnt elicited more severe seizure-like activity. Furthermore, stimulation protocols characterized by high power, long duration, high frequency, and medium pulse width were more prone to inducing such events. Additionally, we found that CA1 stimulation could impair subsequent contextual fear memory. These findings provide critical cautions and practical guidelines for the design and implementation of in vivo optogenetic experiments in neuroscience research.

## Simple Summary

Optogenetics is a widely applied technique in neuroscience that enables the manipulation of neuronal activity with high spatiotemporal precision. However, because optogenetic stimulation can induce artificial neuronal hypersynchronization, the potential risk of triggering seizure-like activity and confounding experimental outcomes remains a concern. Here, we demonstrate through electrophysiological and behavioral recordings that even single-trial optogenetic stimulation is sufficient to induce seizure-like discharges and behaviors, whereas repeated stimulation elicits more severe seizure-like activity. Moreover, stimulation protocols featuring high power, long duration, high frequency, and medium pulse width were more likely to elicit such events and impair ongoing behavioral performance. Our results provide crucial insights and practical guidelines to inform the design and execution of in vivo optogenetic experiments.

## 1. Introduction

High temporal and spatial precision in manipulating neuronal activity via light stimulation is a defining characteristic of optogenetics. It has been widely applied in neuroscience research across diverse animal models, including Drosophila, zebrafish, mice, rats, and nonhuman primates [1,2,3,4]. Opsins, the light-sensitive proteins used in optogenetics, rapidly respond to illumination through unique structural conformational changes, thereby increasing or decreasing cellular activity in vitro or in vivo [3,5]. Consequently, all transduced and illuminated cells become photosensitive and respond simultaneously. This can lead to synchronized neuronal discharges that exceed physiological ranges [3,6]. Because neuronal activities across multiple brain regions are rarely recorded simultaneously in in vivo optogenetic experiments, this potential hypersynchronized effect may have been overlooked. Such hyperactivity could ultimately induce unnatural plasticity within neural circuits [6].

In fact, optogenetics has already been demonstrated to induce seizures because of widespread neuronal hyperexcitability and hypersynchronization (e.g., afterdischarges) [7,8,9,10,11,12,13,14,15]. Optogenetic kindling of neurons in the piriform cortex can disrupt the balance of local transmitter release and affect synaptic plasticity [13]. However, the light stimulation paradigms or parameters (such as power, frequency, pulse width, and duration) used in epilepsy research are often compatible with or partially overlap with those employed in general neuroscience studies [16,17,18]. For instance, activation of CaMKII^+^ neurons in the basolateral amygdala (BLA) with medium light power (7–8 mW) at the fiber tip can induce seizures, whereas milder stimulation (3–5 mW) does not [19]. Nonetheless, light stimulation of equal or even higher power is frequently used in studies investigating functional circuits and behaviors in freely moving animals in vivo [16]. Moreover, unexpected seizure-like activities (such as afterdischarges and behaviors) may be easily overlooked during optogenetic experiments, potentially leading to erroneous conclusions regarding neural circuit function. Therefore, it is crucial to assess whether seizure-like activities are concomitantly induced by in vivo optogenetic manipulations and to evaluate their potential influence on ongoing task performance.

In this study, we investigated the electrophysiological and behavioral effects of light stimulation across various brain regions using a range of commonly used parameters in freely moving mice. We found that even a single trial of light stimulation could induce seizure-like activities. Specifically, the severity and susceptibility of seizures increased with higher stimulation repetition, intensity, frequency, or duration, as well as with medium pulse widths, significantly influencing ongoing task performance in mice. These results suggest that the risk of inducing seizures should be taken into consideration during paradigm design and data interpretation when employing in vivo optogenetics in neuroscience research.

## 2. Materials and Methods

### 2.1. Animals

The surgical and experimental procedures were approved in advance and conducted in accordance with the guidelines of the Animal Care and Use Committees at the Shenzhen Institutes of Advanced Technology or the Innovation Academy for Precision Measurement Science and Technology, Chinese Academy of Sciences. Adult (2–4 months) male C57BL/6 mice (weighing 20–25 g) were purchased from Hunan SJA Laboratory Animal Company. The animals were provided with food and water ad libitum and housed in a dedicated room with a 12/12 h light/dark cycle.

### 2.2. Virus Information

AAV-CaMKII-ChR2 (H134R)-mCherry (titer: 5 × 10^12^ vg/mL) was purchased from Heyuan Bio Co., Ltd. (Shanghai, China). AAV-CaMKII-mCherry (titer: 4 × 10^12^ vg/mL) was purchased from Braincase Co., Ltd. (Wuhan, China). According to a previous study, the percentage of co-expression of CaMKII+ and ChR2 is very high, all viruses were divided into small aliquots and stored at −80 °C for later use.

### 2.3. Virus Injection and Optical Fiber/Electrode Implantation

The mice were anesthetized with sodium pentobarbital (80 mg/kg) and placed on a stereotaxic apparatus (RWD, 68030, Shenzhen, China). The skull above the target area was thinned using a dental drill (STRONG, Shenzhen, China) and carefully removed with a curved needle. The virus (100 nL) was injected into the target brain regions (field CA1 of the hippocampus (CA1): A–P, −1.70 mm; M–L, −1.56 mm; D–V, −1.05 mm, 14° from the horizontal axis, *n* = 68 mice; anterior piriform cortex (APC): A–P, 1.40 mm; M–L, −2.65 mm; D–V, −4.60 mm, *n* = 7 mice; lateral entorhinal cortex (LEnt): A–P, −3.20 mm; M–L, −4.20 mm; D–V, −4.30 mm, *n* = 8 mice; medial entorhinal cortex (MEnt): A–P, −4.80 mm; M–L, −3.60 mm; D–V, −3.80 mm, *n* = 5 mice; basolateral amygdaloid nucleus, anterior part (BLA): A–P, −1.55 mm; M–L, −3.35 mm; D–V, −4.70 mm, *n* = 4 mice; central amygdaloid nucleus (CEA): A–P, −1.45 mm; M–L, −2.95 mm; D–V, −4.40 mm, *n* = 5 mice; ventromedial hypothalamic nucleus (VMH): A–P, −1.50 mm; M–L, −0.50 mm; D–V, −5.60 mm, *n* = 3 mice; primary visual cortex (V1): A–P, −3.20 mm; M–L, −2.50 mm; D–V, −0.90 mm, *n* = 3 mice; lateral septal nucleus (LS): A–P, 0.50 mm; M–L, −0.50 mm; D–V, −3.20 mm, 10° from the horizontal axis, n = 3 mice; paraventricular hypothalamic nucleus (PVN): A–P, −0.86 mm; M–L, −0.20 mm; D-V, −5.05 mm, *n* = 5 mice) using a microinjector connected to a glass micropipette (WPI, 4878, Sarasota Bay, FL, USA) and a syringe pump (Stoelting, Quintessential Stereotaxic Injector, 53311, Wood Dale, IL, USA). The number of mice used in each group was estimated based on our preliminary exploratory data. After injection, the micropipette was left in place for 10 min before being slowly withdrawn. An optical fiber (numerical aperture: 0.22, diameter: 230 μm) was inserted into the defined region so that the tip of the fiber resided 0.25 mm above the injection site. Electrodes were implanted bilaterally into the dentate gyrus (DG) (A-P, −1.70 mm; M-L, ±0.90 mm; D-V, −1.95 mm), BLA, APC, or posterior piriform cortex (PPC) (A-P, −1.10 mm; M-L, −3.50 mm; D-V, −5.60 mm). Two stainless steel screws were inserted above the cerebellum to serve as ground and reference electrodes. Virus injection and optical fiber/electrode implantation sites were carefully verified, and only data from subjects with injection sites strictly restricted to the target brain regions were included in the analysis. Mice were allowed to recover from surgery for at least 3 weeks, and all efforts were made to minimize animal suffering and distress. A total of 111 mice were used in this study, and data from 8 mice were excluded because the injection sites were outside the target brain regions. In accordance with the 3R principles, certain mice were reused across experiments to minimize animal usage while fulfilling the study objectives.

### 2.4. Photostimulation

#### 2.4.1. Single-Trial Light Stimulation

After 3 weeks of AAV expression, mice were placed in a transparent box (25 × 25 × 25 cm) and habituated for 10 min. Subsequently, a single-trial light stimulation (20 Hz, 5 ms, 10 s), controlled by a Master-8 stimulator (AMPI, Jerusalem, Israel), was administered. Light power was set to 3 mW at the fiber tip from a fiber-coupled diode laser (473 nm) to activate ChR2-expressing neurons.

#### 2.4.2. Repeated Light Stimulation

Mice received four trials of light stimulation (20 Hz, 5 ms, 10 s) with a 5-min interval on the first day, delivered at a light power of 3 mW at the fiber tip using a fiber-coupled diode laser (473 nm). On the second and sixteenth days, mice received identical light stimulation protocols. For APC-LEnt projection activation, mice received four trials of light stimulation (20 Hz, 5 ms, 15 s) with a 5-min interval for four consecutive days, using a light power of 5 mW at the fiber tip.

### 2.5. Light Parameter Tests

#### 2.5.1. Power

Mice received consecutive light stimulations (20 Hz, 5 ms, 10 s) with a 2-min interval and gradually increasing power (0.05, 0.1, 0.2, 0.5, 1, 2, 3, and 5 mW at the fiber tip). After the threshold power (defined as the power at which the first ADs are induced, keeping stimulation frequency, pulse width, and duration constant) was determined, the interval between consecutive stimulations was increased to 10 min to abate any post-ictal refractory effects. This principle was applied to all light parameter test experiments.

#### 2.5.2. Frequency

Mice received consecutive light stimulation (5 ms, 10 s, 3× threshold power) with a 2-min interval and gradually increasing frequency (1, 5, 10, 20, and 40 Hz).

#### 2.5.3. Pulse Width

Mice received consecutive light stimulations (20 Hz, 10 s, 3× threshold power) with a 2-min interval and gradually increasing pulse width (1, 5, 10, 15, and 20 ms).

#### 2.5.4. Duration

Mice received consecutive light stimulations (20 Hz, 5 ms, 3× threshold power) with a 2-min interval and gradually increasing duration (0.5, 1, 2, 4, 6, 8, 10, 20, and 60 s).

### 2.6. Electrophysiological Recordings

Local field potentials (LFPs) and electroencephalographies (EEGs) were recorded using an amplifier (Model 3000, A-M Systems, Washington, DC, USA; amplified 100×), filtered (1–300 Hz), and digitized at 1 kHz (Power 1401, Cambridge Electronic Design, Cambridge, UK) using Spike2 (version 6.06) software. Two cameras (top view and side or bottom view) were used to simultaneously record video for behavioral analysis. To minimize bias, electrophysiological and video recordings were evaluated in a blinded manner by assessors who were unaware of the group allocations.

### 2.7. Behavioral Analysis

#### 2.7.1. Seizure-like Behaviors

Seizure-like behaviors during and after light stimulation were classified according to a modified Racine scale [13,20]: 0, normal behavior; 1, freezing and facial movement; 2, head nodding; 3, unilateral forelimb clonus; 4, bilateral forelimb clonus and rearing; 5, rearing and falling; and 6, running and jumping seizures. Freezing and head nodding occurring during or after light stimulation and coinciding with electrophysiological ADs were defined as seizure-like behaviors.

#### 2.7.2. Contextual Fear Conditioning

On the first day (day 1), mice were placed in a chamber (18 × 18 × 25 cm^3^, with a grid floor) and habituated for 2 min, followed by a 20-s blue light stimulation (20 Hz, 5 ms) that co-terminated with a footshock (2 s, 0.5 mA). Mice were returned to their home cages 3 min after the termination of stimulation. On the second day (day 2), mice were placed back in the chamber for a 5-min conditioned fear retention test and subsequently returned to their home cages.

#### 2.7.3. Slice Preparation and Imaging

Mice were anesthetized with an overdose of sodium pentobarbital (120 mg/kg) and transcardially perfused with phosphate-buffered saline (PBS) followed by 4% paraformaldehyde (PFA, Sigma-Aldrich, 158127MSDS, St. Louis, MO, USA). Brains were extracted and post-fixed overnight in 4% PFA at 4 °C. Coronal sections (40 μm thick) were cut using a cryostat microtome (Thermo Fisher, NX50, Waltham, MA, USA). Sections were stained with 4′, 6-diamidino-2-phenylindole (DAPI), mounted in 70% glycerol, and visualized using a confocal microscope (Leica, TCS SP8, Buffalo Grove, IL, USA) or a virtual microscopy slide-scanning system (Olympus, VS 120, Tokyo, Japan).

### 2.8. Immunohistochemistry

The sections were washed with PBS (3 × 5 min) and incubated in a blocking solution (10% normal goat serum and 0.3% Triton X-100 in PBS) for 1 h at 37 °C, followed by primary antibody incubation with anti-c-Fos (Cell Signaling Technology, Danvers, MA, USA; 2250S, 1:700) for 48 h at 4 °C. After washing with PBS (3 × 10 min), the sections were incubated with goat anti-rabbit FITC (Jackson ImmunoResearch, West Grove, PA, USA; 94600, 1:200) for 1 h at 37 °C, washed again with PBS (3 × 10 min), stained with DAPI, and mounted in 70% glycerol.

### 2.9. Data Analysis

#### 2.9.1. Estimation of Seizure-like ADs

Light stimulation-induced ADs were defined as LFP spike waves with interspike intervals within a 1–10 Hz range, a mean voltage exceeding the baseline plus 3 times standard deviations, and a duration longer than 3 s. The latency to AD onset was measured as the time elapsed from the initiation of light stimulation to the first appearance of AD activity.

#### 2.9.2. Freezing Rate and Velocity

The freezing rate (day 2) and averaged velocity (day 1, measured before and after light stimulation and footshock) were analyzed using EthoVision XT (Noldus Information Technology, Wageningen, The Netherlands).

#### 2.9.3. Statistical Analyses

**Figure 1:**
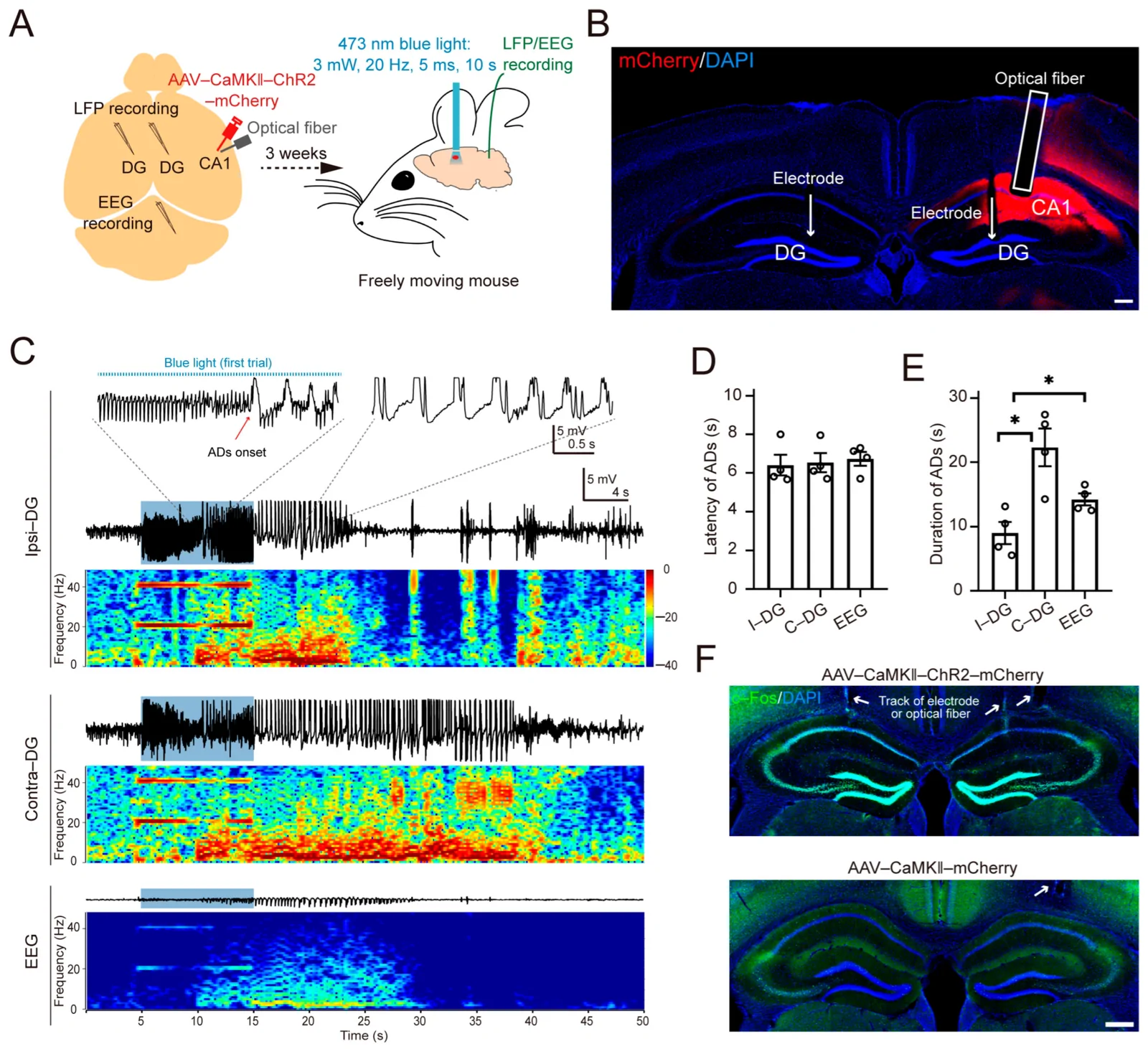
Single-trial optogenetic activation of CA1 CaMKII^+^ neurons induces seizure-like activity. (**A**). Schematic of viral injection, optical fiber/electrode implantation, and recording in freely moving mice. Optogenetic stimulation parameters: power, 3 mW (at fiber tip); frequency, 20 Hz; pulse width, 5 ms; duration, 10 s. (**B**). Representative images showing viral injection and light stimulation in the unilateral CA1, alongside local field potential (LFP) recording sites in the bilateral DGs. Scale bar: 200 μm. Nuclei were stained with DAPI. (**C**). LFP/EEG traces (top) and corresponding time–frequency spectrogram (bottom). Seizure-like afterdischarges (ADs) were evoked by optogenetic stimulation and recorded in the bilateral DGs (upper and middle panels) and the cerebellum (lower panels). Blue shading denotes light stimulation; the red arrow indicates the onset of ADs. (**D**,**E**). Quantification of AD latency (**D**) and duration (**E**). *n* = 4 mice/group; * *p* < 0.05. Statistical analyses were performed using a one-way RANOVA followed by Bonferroni test. Data are presented as mean ± SEM. (**F**). Representative immunofluorescent images showing c-Fos expression (green) following light stimulation. Diagonal arrows indicate the electrode or optical fiber tracks. Nuclei were stained with DAPI. Scale bar: 200 μm.

Differences in the latency and duration of seizure-like activities (Figure 1D,E) were evaluated using one-way RANOVA followed by Bonferroni test. Averaged daily Racine scores were analyzed using Friedman test followed by post hoc Wilcoxon signed-rank tests. Differences in the freezing rate were assessed using one-way ANOVA followed by LSD post hoc tests and FDR correction; square root-transformed (SQRT) data for these measurements are presented. All statistical analyses were performed using SPSS (22.0, IBM Corp., Armonk, NY, USA). Statistical significance was set at * *p* < 0.05, ** *p* < 0.01 and *** *p* < 0.001. All values are presented as the mean ± standard error of mean (SEM). Graphs were generated using GraphPad Prism 8 (GraphPad Software, San Diego, CA, USA).

## 3. Results

### 3.1. Single-Trial Optogenetic Activation of Neurons Induces Seizure-like Activity

To determine whether optogenetic activation can induce seizure-like activity during behavioral and cognitive studies, we applied a commonly used stimulation paradigm to freely moving mice. We transduced ChR2 (H134R) to CaMKII^+^ neurons by injecting AAV-CaMKII-ChR2-mCherry into the right CA1. After 3 weeks, a single trial of blue light stimulation (3 mW, 20 Hz, 5 ms pulse width, 10 s duration) was delivered to the CA1. LFPs from bilateral DGs and EEGs from the cerebellum were recorded simultaneously in freely moving mice (Figure 1A,B). Strikingly, this single optogenetic stimulation elicited robust, long-lasting seizure-like ADs (Figure 1C). Concurrently, subtle seizure-like behaviors, such as freezing or head nodding, were also observed (Table 1). Notably, a single trial of light stimulation, rather than repeated or consecutive sessions, was sufficient to induce ADs. All mice in this study were naïve and had no prior exposure to optogenetic stimulation. Furthermore, ADs emerged prior to the termination of the light pulse, indicating that the duration of light activation was more than adequate. Time-frequency analysis revealed a robust increase in LFP and EEG power within the low-frequency band (1–20 Hz, Figure 1C). Further analysis demonstrated that while the onset latency of ADs was consistent across the recorded brain regions, their duration varied significantly (Figure 1D,E). Immunohistochemical staining showed that c-Fos signals were not only enriched at the recording sites (Figure 1F), but also widespread across multiple brain regions, including the anterior cingulate cortex (ACC), lateral septal nucleus (LS), bed nucleus of the stria terminalis (BNST), piriform cortex (PC), amygdala, ventral hippocampus (vHPC), ventromedial hypothalamic nucleus (VMH), paraventricular hypothalamic nucleus (PVN), lateral hypothalamic area (LHA), and entorhinal cortex (Ent) (Figure S1). Collectively, these data support extensive neuronal activation across widespread brain networks associated with seizure-like ADs induced by single-trial optogenetic activation of CA1 CaMKII^+^ neurons. Because light delivery can potentially heat brain tissue and alter neuronal firing [21,22], we investigated whether laser heating could induce seizure-like activity. We injected AAV-CaMKII-mCherry into the CA1 as a control (Figure S2A,B) and applied the same stimulation protocol. No discernible ADs or seizure-like behaviors were observed during or after light stimulation (Figure S2C). These results indicated that the seizure-like activities induced by optogenetic stimulation were attributed to the specific activation of ChR2-expressing neurons rather than photothermal effects.

**Figure 2:**
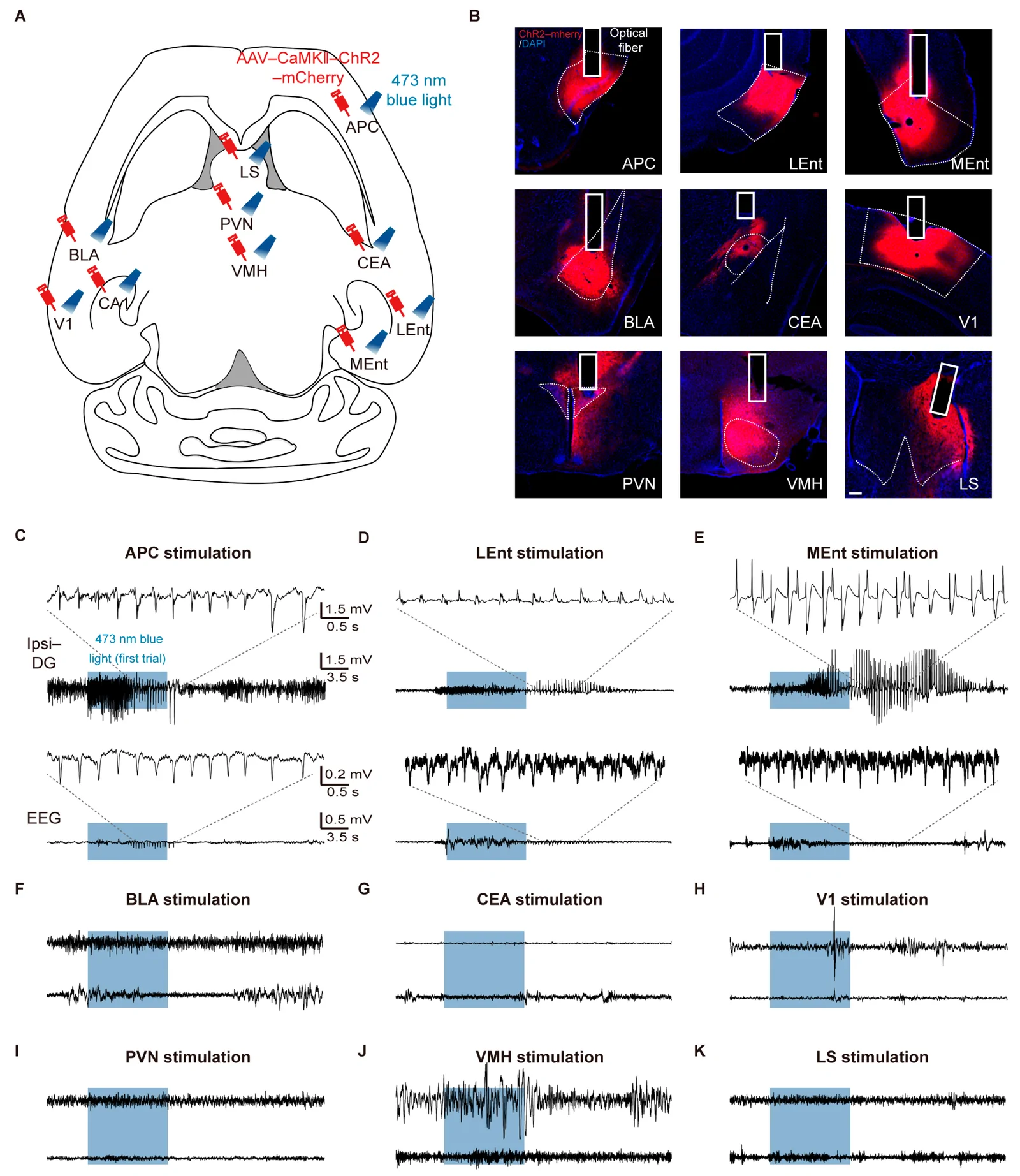
Single-trial optogenetic activation of CaMKII^+^ neurons across distinct brain regions produces differential effects on seizure induction. (**A**). Schematic of in vivo optogenetic experiments targeting different brain regions. Optogenetic activation was applied to each brain region separately. Blue shading indicates the period of light stimulation. (**B**). Representative images showing viral injection and light stimulation sites. White box: optical fiber track. Scale bar: 200 μm. (**C**–**K**). Representative LFP/EEG traces during and after single-trial light stimulation in different brain regions. Robust ADs were detected in the APC (**C**), LEnt (**D**) and MEnt (**E**) stimulation groups. Blue shading indicates light stimulation.

To investigate whether the induction of seizure-like activity is specific to the CA1, we performed analogous experiments in other brain regions. We injected AAV-CaMKII-ChR2-mCherry into the anterior piriform cortex (APC), lateral Eent (LEnt), medial Ent (MEnt), anterior portion of the basolateral amygdaloid nucleus (BLA), central amygdaloid nucleus (CEA), primary visual cortex (V1), PVN, VMH and LS in separate cohorts of mice (Figure 2A,B). Consistent with the findings in the CA1, optical activation of the APC, LEnt and MEnt elicited a high rate of ADs (Figure 2C–E, Table 1) accompanied by mild seizure-like behaviors, such as freezing or head nodding. In contrast, no ADs or seizure-like behaviors were observed in mice with activation of the BLA, CeA, V1, PVN, VMH, or LS (Figure 2F–K, Table 1).

Collectively, these results indicate that single-trial optogenetic activation of CaMKII^+^ neurons across multiple brain regions can elicit large-scale, long-lasting seizure-like ADs and associated behaviors in mice.

### 3.2. Repetitive Optogenetic Stimulation Enhances Seizure Susceptibility

**Figure 3:**
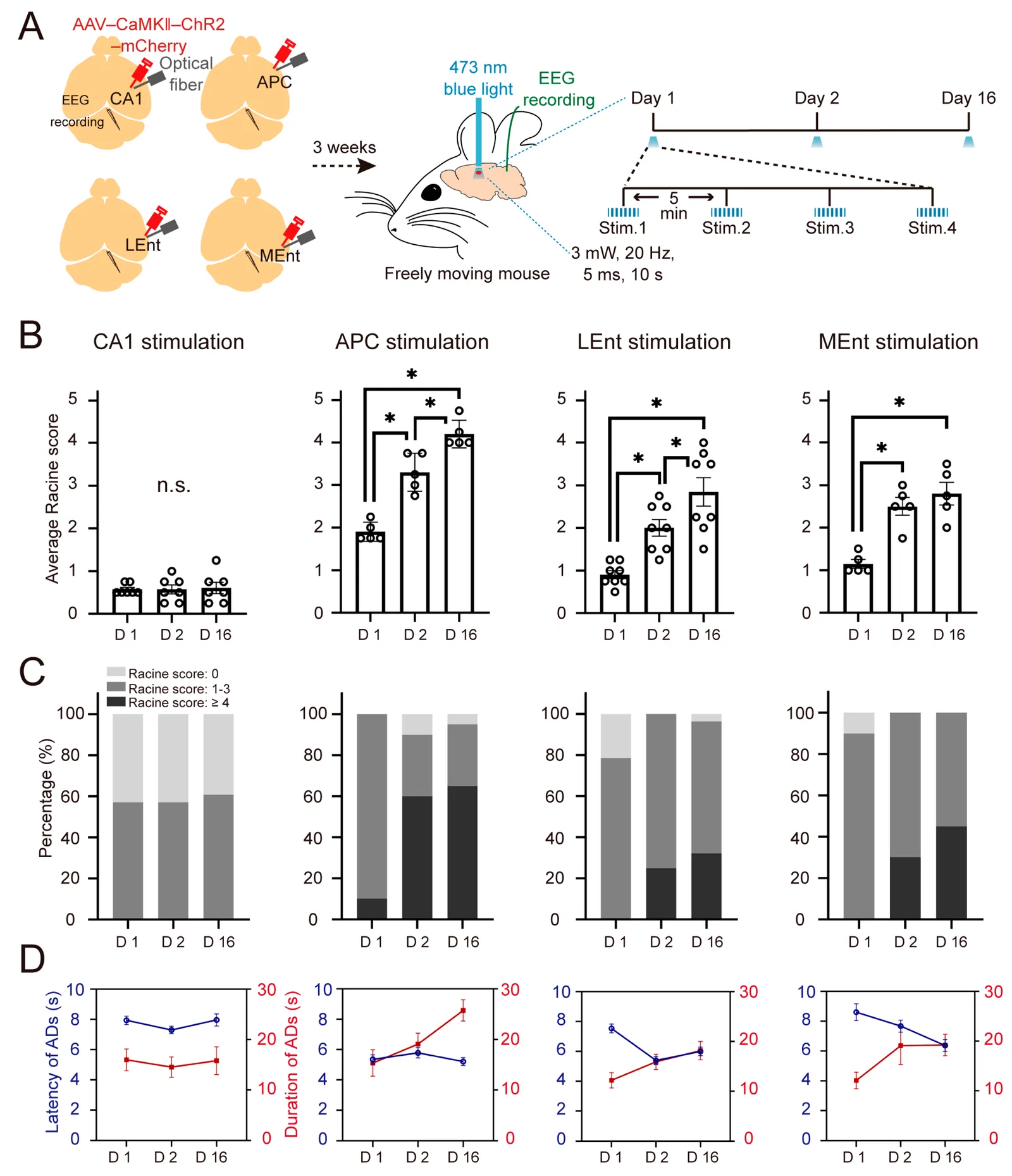
Repetitive optogenetic stimulation in specific brain regions increases seizure susceptibility. (**A**): Schematic of the in vivo repetitive optogenetic stimulation protocol. Virus was injected into the CA1, APC, LEnt and MEnt, respectively. After three weeks, mice received trains of light pulses. Each stimulation train was delivered four times per day (Stim.1–Stim.4), and repeated on days 1, 2 and 16. Light stimulation parameters: power, 3 mW (at the optical fiber tip); frequency, 20 Hz; pulse width, 5 ms; duration, 10 s. (**B**): Mean daily Racine scores averaged across four daily stimulation trials on days 1, 2 and 16 for the four experimental groups. *n* = 7 (CA1), *n* = 5 (APC), *n* = 8 (LEnt), *n* = 5 (Ment) mice; * *p* < 0.05, n.s., no significant difference. Friedman test followed by post hoc Wilcoxon signed-rank tests was performed. Data are presented as mean ± SEM. (**C**): Percentage distribution of each Racine score on days 1, 2 and 16 in the four experimental groups. Racine score 0, no seizure behavior; scores 1–3, focal seizures; score ≥ 4, generalized seizures. (**D**): Mean latency and duration of ADs averaged across four daily stimulation trials on days 1, 2 and 16 for the four groups.

Repetitive light stimulation is widely used in studies of learning, memory, emotion, and clinical therapeutics [1,23]. However, whether repetitive optogenetic activation of neurons or their projections exacerbates seizure severity or susceptibility remains unclear. To address this, we injected AAV-CaMKII-ChR2-mCherry into the CA1, APC, LEnt, and MEnt, respectively. ChR2-expressing neurons were then activated using light pulse trains administered four times daily for two consecutive days (Figure 3A). On day 1, mice exhibited mild focal seizures (indicated by low average Racine scores) in response to stimulation across four targeted regions (Figure 3B). On day 2, mice showed more severe seizure-like behaviors with significantly higher average Racine scores following activation of the APC, LEnt, or MEnt, but not the CA1 (Figure 3B). These data demonstrate that repetitive light activation of CaMKII^+^ neurons in the APC, LEnt, or MEnt enhances seizure-like activity.

To further evaluate the persistence of this enhanced seizure susceptibility, we repeated the stimulation protocol two weeks later (day 16) (Figure 3A). Light stimulation induced significantly more robust seizure behaviors in mice with APC, LEnt, or MEnt activation, characterized by higher average Racine scores and an increased percentage of generalized seizures (Racine score ≥ 4) In contrast, the CA1 group showed no such enhancement. (Figure 3B,C). Consistent with the behavioral data, EEG recordings revealed that the duration of ADs was markedly prolonged in the APC, LEnt, or MEnt groups, but only modestly altered in the CA1 group. Furthermore, the latency to AD onset was significantly reduced in the LEnt or MEnt groups, whereas changes in the CA1 and APC groups were minimal (Figure 3D). Collectively, these results indicate that the susceptibility to optogenetically induced seizures does not diminish over time rather, it remains significantly elevated two weeks after the initial stimulation.

To assess the cumulative effect of individual optogenetic stimulation trials, we analyzed seizure-like activity across the three time points. The data demonstrate that prior optogenetic stimulation generally enhances the seizure-inducing efficacy of subsequent trials in mice with APC, LEnt, or MEnt activation, but not in those with CA1 activation (Figure S3).

Because single-trial light stimulation can induce seizure-like activity in the CA1, APC, LEnt, and MEnt, and repetitive stimulation enhances seizure susceptibility, we sought to determine whether repetitive stimulation could induce seizures in less sensitive regions. We applied the aforementioned repetitive light stimulation protocol to the BLA (Figure S4A), which exhibited no ADs following the initial light stimulation on day 1. Unexpectedly, mild ADs were elicited by the first trial on day 2, and robust AD were induced two weeks later by the first trial (Figure S4B). These data indicate that repetitive light stimulation can induce and potentiate seizure-like activity, even in brain regions that are initially insensitive.

Optogenetic stimulation targeting specific neuronal projections has been widely used to study functional connectivity across distinct brain regions [2]. To investigate whether such optogenetic manipulation of specific neuronal projections evokes seizure-like activity, we injected AAV-CaMKII-ChR2-mCherry into the APC and administered light stimulation to the LEnt in four mice (Figure S5A,B). Our preliminary results showed that single-trial light stimulation elicited typical ADs within the ipsilateral DG in two mice (Figure S5C), indicating that single-trial optogenetic activation of the APC-LEnt projection may induce seizure-like activity. We then activated this neuronal projection with repetitive light stimulations as described above. Although the daily average Racine scores of the experimental mice increased, no statistically significant difference was detected between day 2 and day 4 (Figure S5D,E). Collectively, these results suggest that repetitive light stimulation of specific neuronal projections may heighten seizure susceptibility in mice.

### 3.3. Relationship Between Light Stimulation Parameters and Seizure Occurrence

**Figure 4:**
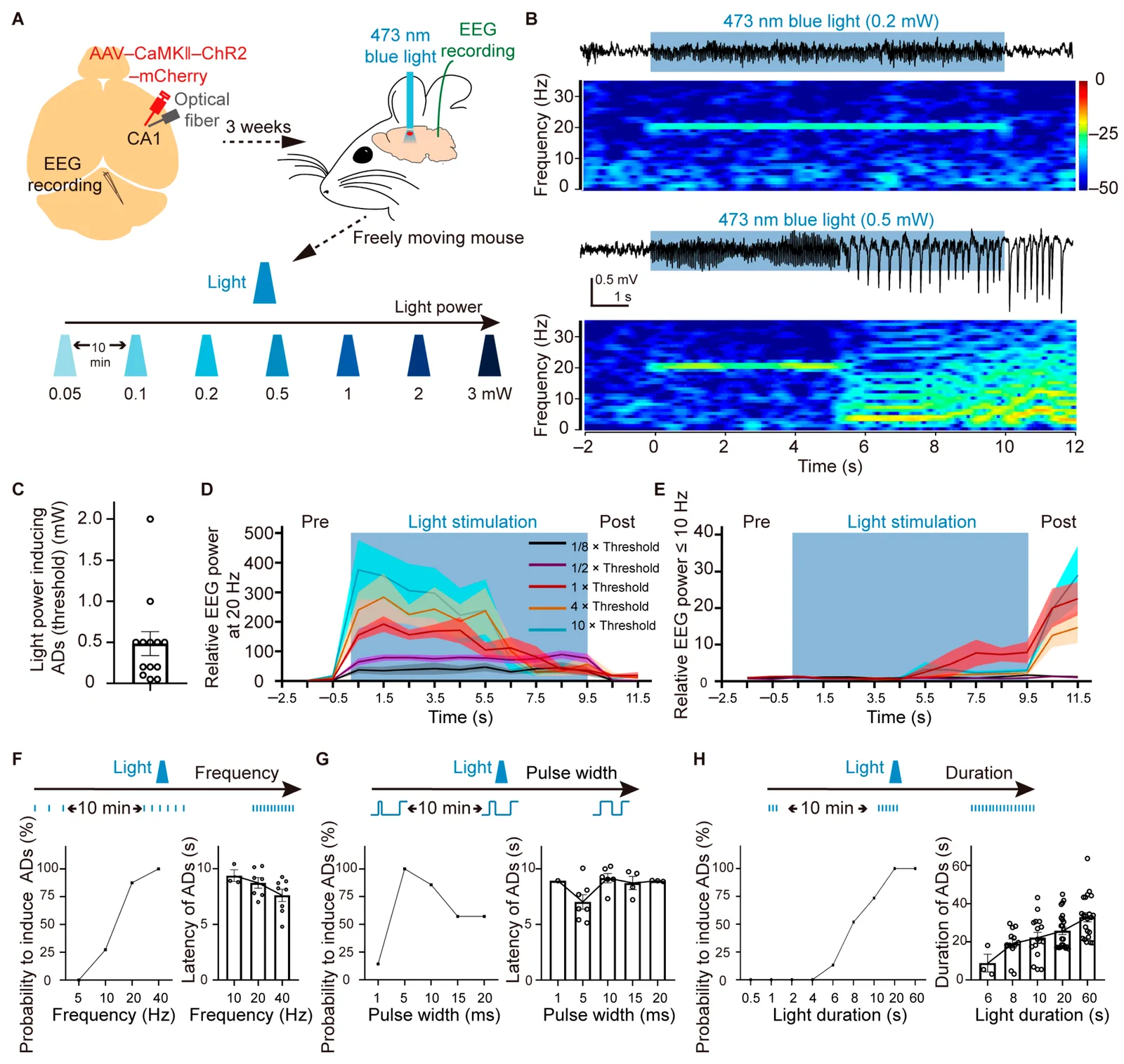
Relationship between light stimulation parameters and seizure induction. (**A**): Experimental schematic of viral injection, light stimulation and EEG recording. Virus was injected into the unilateral CA1. After three weeks, light pulses were delivered with increasing power (0.05–5 mW) while frequency, pulse width, and duration were held constant (20 Hz, 5 ms, 10 s). (**B**): Representative raw EEG traces (top) and corresponding time–frequency spectrograms (bottom). ADs were not evoked at low light intensity (0.2 mW, upper panel) but were reliably induced at slightly higher intensity (0.5 mW, lower panel). (**C**): Threshold light power required to elicit the first AD as power was gradually increased (*n* = 13 mice). (**D**): Relative EEG power spectrum at 20 Hz. EEG power at 20 Hz (evoked by light stimulation) increased with rising light intensity. (**E**): Relative EEG power spectrum at ≤10 Hz. EEG power in the ≤10 Hz band (corresponding to ADs) increased only during suprathreshold stimulation trials. (**F**): Higher frequency light stimulation more effectively elicits ADs. Increasing stimulation frequency (5–40Hz) was associated with a higher probability of AD induction (left panel) and a shorter onset latency (right panel). *n* = 8 mice. (**G**): Medium pulse-width light stimulation preferentially induces ADs. Stimulations with a medium pulse width (5 ms) produced a higher AD induction probability (left panel) and a shorter onset latency (right panel). *n* = 7 mice. (**H**): Long duration light stimulation more effectively induces ADs. The AD induction probability increased with longer light duration (left panel), and the duration of evoked ADs also increased with increasing light stimulation duration (right panel). *n* = 23 mice. Data are presented as mean ± SEM.

Light stimulation parameters, including power, frequency, and pulse width, differ across neuroscience studies [5,16]. Accordingly, it is necessary to determine whether seizure-like activity can be triggered during optogenetic experiments using conventionally adopted parameter ranges. To clarify the correlation between light stimulation parameters and seizure induction, we chose the CA1 for optogenetic activation, as it exhibited stable ADs and mild seizure behaviors without exacerbation following repeated light stimulation. First, we activated CaMKII^+^ neurons in the CA1 by gradually increasing light power from 0.05mW to 5 mW while keeping the frequency (20 Hz), pulse width (5 ms), and stimulation duration (10 s) constant (Figure 4A). No ADs were observed until the light power reached a specific threshold (Figure 4B); a low-to-moderate power level widely used for in vivo optogenetic experiments (0.48 ± 0.15 mW, *n* = 13 mice) (Figure 4C). Quantitative analysis showed that the 20 Hz EEG power in response to light stimulation increased during light delivery. In suprathreshold stimulation trials, the 20 Hz EEG power rapidly increased right after light stimulation onset, maintained a high, stable level, and then declined before the termination of stimulation. However, in subthreshold stimulation trials, the 20 Hz EEG power only slightly rose and remained stable throughout stimulation (Figure 4D). Regarding low-frequency EEG power (≤10 Hz, mainly reflecting ADs), no obvious change occurred at the onset of light stimulation. A sustained increase in this frequency band was observed only during and after suprathreshold stimulation trials (Figure 4E).

We further investigated how light stimulation frequency and pulse width affect seizure induction. The results showed that high-frequency (40 Hz) and medium-pulse-width (5 ms) light stimulation tended to increase AD probability and reduce AD latency. (Figure 4F,G). Of note, low-frequency (1 or 5 Hz) light stimulation did not induce ADs. As described above, ADs typically emerged before the termination of light stimulation. It remained unclear whether short-duration light stimulation could prevent seizure induction. To address this, we activated CA1 CaMKII^+^ neurons by gradually prolonging the stimulation duration (Figure 4H). We found that light stimulation with a duration of ≤4 s could not trigger seizures. Nevertheless, when the stimulation duration exceeded 6 s, both AD probability and AD duration showed a tendency to increase accordingly. These results demonstrate that light stimulation characterized by high power, long duration, high frequency, and medium pulse width appeared to be more likely to induce seizures.

Because long-term ChR2 expression is commonly employed to investigate neural circuit function [24], we assessed its effects on seizure induction. We injected AAV-CaMKII-ChR2-mCherry into the CA1 and performed optogenetic experiments 6 weeks post-injection (Figure S6A). Relative to neuronal activation measured 3 weeks after AAV injection, light stimulation of CA1 CaMKII^+^ neurons at 6 weeks evoked seizures under similar light parameters (threshold power, duration, frequency, and pulse width) (Figure S6B–E). These results indicate that long-term ChR2 expression has little to no effect on seizure susceptibility.

### 3.4. Optogenetic Stimulation Induced ADs Associated with Impaired Fear Memory

**Figure 5:**
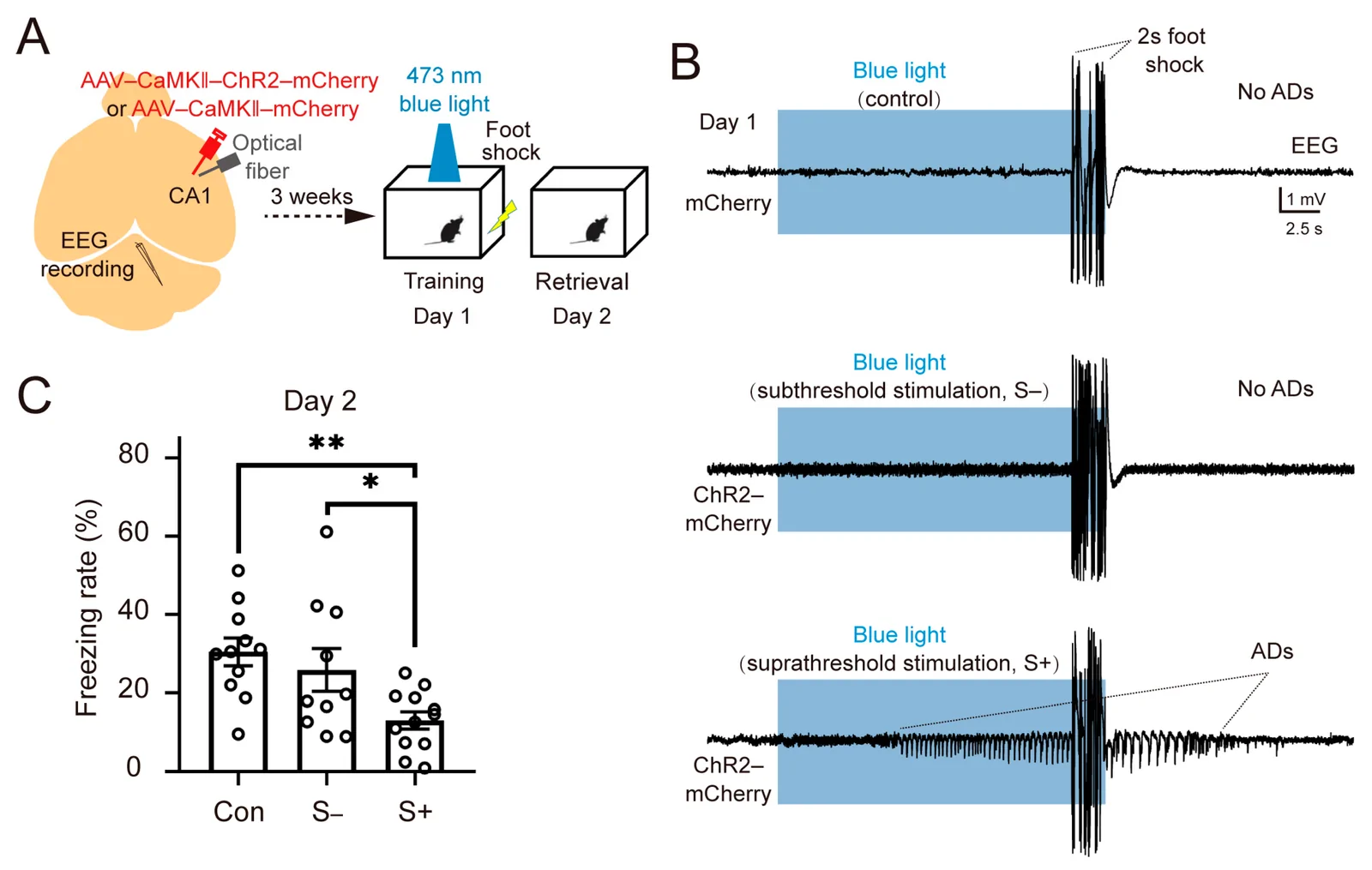
ADs induced by optogenetic stimulation associated with impaired contextual fear memory. (**A**): Experimental schematic. Virus was injected into the CA1 region. After three weeks, mice underwent fear conditioning on day 1, followed by a freezing behavior test on day 2. During day 1 conditioning, a 20 s light stimulation was applied such that it terminated simultaneously with a 2 s footshock stimulus, and real-time EEG recordings were performed throughout this process. (**B**): Representative raw EEG traces during light stimulation. Suprathreshold stimulation induced ADs (bottom), whereas no ADs were observed in the control (top) or subthreshold-stimulation (middle) groups. Of note, high-amplitude signals appearing within the 2-s footshock window were movement artifacts generated when mice attempt to escape the footshock). (**C**): Quantification of freezing percentages across experimental groups on day 2. Mice receiving suprathreshold stimulation that induced ADs exhibited a significantly reduced freezing time. Statistical analyses were performed using a one-way ANOVA followed by LSD post hoc tests and FDR correction (Control group, *n* = 11 mice; S^−^ group, *n* = 10 mice; S^+^ group, *n* = 12 mice). * *p* < 0.05, ** *p* < 0.01. Data are presented as mean ± SEM.

The CA1 is a core brain region responsible for learning and memory [25]. Previous studies have shown that optogenetic activation of CA1 CaMKII^+^ neurons (20 Hz, 15 ms, 12 mW, 40 s or 20 Hz, 20 ms, 4–9 mW, 30 s) disrupts contextual fear conditioning [26,27]. Nevertheless, a separate study using parameters of 20 Hz, 15 ms, 8–10 mW for 30 s, reported no such memory impairment [28]. Such inconsistent findings may arise from light-evoked ADs, which disrupt animal behavior. To validate this hypothesis, we adopted a contextual fear conditioning paradigm in our experiment (Figure 5A). We determined the threshold light power for each mouse before conducting the contextual fear conditioning test. On day 1, mice were randomly assigned to two experimental groups: suprathreshold stimulation [S^+^] and subthreshold stimulation [S^−^]. (The suprathreshold and subthreshold stimulation levels we used were 300% and 1/3 of the threshold, respectively.). All mice received a 20s (20 Hz, 5 ms pulse width) light delivery that co-terminated with a 2s footshock. Mice expressing only mCherry served as controls and received matching light stimulation and footshocks. EEG data showed that the stimulation elicited ADs in the S^+^ group, whereas no ADs were detected in the S^−^ or control groups (Figure 5B). On day 2, conditioned fear responses were tested without stimulation. The S^+^ group exhibited a significantly lower freezing rate relative to both the S^−^ and control groups, whereas no statistical difference in freezing behavior was observed between the S^−^ and control groups (Figure 5C). These results suggest that optogenetic activation of CA1 CaMKII^+^ neurons accompanied by ADs was associated with impaired subsequent contextual fear memory.

## 4. Discussion

Optogenetics is a powerful tool that allows precise regulation of activity within genetically defined neuronal circuits in freely behaving animals, enabling researchers to decipher causal relationships between neural activity and behavior [1]. However, synchronous excitation or inhibition of neuronal populations by optogenetic stimulation can produce neuronal hypersynchrony that is rarely observed under physiological conditions [6]. It is critical to characterize potential side effects of optogenetic stimulation, such as seizure-like discharges and behaviors, and to investigate how these events affect animal behavior and physiology during and after optogenetic stimulation in freely behaving mice. Such efforts will facilitate more rigorous application of optogenetic tools. Moreover, elucidating how these optogenetic side effects depend on light parameters is critically important.

To address this question, in the present study, we expressed ChR2 in CaMKII^+^ neurons of target brain regions, and delivered light stimulation to these regions individually, while simultaneously recording neural activity from bilateral DGs and the cerebellum. We found that a single trial of optogenetic activation in the CA1, APC, LEnt, or MEnt alone was able to induce typical seizure-like ADs and behaviors in mice. Notably, the light parameters we used were equivalent to or even lower than those commonly reported [16], These results are partially consistent with prior research showing that prolonged 20Hz blue light stimulation of APC neurons can induce seizures in mice [14,17]. This implies that seizure-like ADs and behaviors can be easily elicited by optogenetic stimulation in specific brain regions, a critical artifact that has been overlooked in past work.

We then delivered repeated light stimulations to the selected brain regions and found that optogenetic activation in the APC, LEnt, or MEnt led to more severe seizures, supporting the idea that repeated optogenetic activation can serve as a novel kindling method [9,12,13,15]. This parallels classical electrical or chemical stimulation, where repeated stimulations (electric or chemical kindling) of the HPC, PC and Ent have been established as effective kindling models characterized by seizure-like EEGs and behaviors [11,29,30,31,32,33]. For example, daily electrical stimulation of subcortical nuclei in rats can elicit motor seizures [34], and repeated electrical stimulation of certain brain areas can decrease the threshold for epileptiform ADs while increasing AD duration (Racine 1972) [20]. In addition, we found that optogenetic activation of the APC—LEnt projection can induce ADs, consistent with previous findings [17]. Because seizure-like activities induced by optogenetic activation of a neuronal projection are rarely reported, these results indicate that optogenetic stimulation may induce seizures regardless of whether cell bodies or axon terminals are targeted.

Moreover, by systematically evaluating optogenetic stimulation parameters within commonly used ranges, we observed a tendency for mice to be more prone to light-evoked seizure induction under conditions of high laser power, high stimulation frequency, and medium pulse widths (5–10 ms). This result aligns with prior electrical stimulation study showing that higher frequencies (100 vs. 50 Hz), broader pulse widths (1 vs. 0.2 ms) [35], shorter intertrial intervals, or longer stimulation durations [36] produce more robust AD induction. In this study, we further demonstrated that suprathreshold CA1 stimulation accompanied by ADs was associated with impaired subsequent contextual fear memory in mice. These findings indicate that seizure activity substantially alters the brain’s physiological state, which in turn severely impairs performance on concurrent behavioral tasks. Collectively, our results suggest that light-evoked seizure-like activity may represent a potentially important confounding factor under certain commonly used stimulation conditions. Because overt seizure manifestations, such as freezing and immobility, are subtle and often overlooked during behavioral assessment, our findings serve as a cautionary note for ensuring rigorous behavioral data analysis in optogenetic studies.

Notably, given that the AD induction rate in highly susceptible brain regions, such as CA1 and APC, exceeds 80%, the absence of ADs in 3–5 mice within regions like the CEA, BLA, V1, VMH, PVN, and LS suggests that these areas may exhibit lower susceptibility, though the possibility of a type II error cannot be excluded. It is likely associated with distinct neuronal populations targeted by CaMKII-promoter-driven viral transduction within these areas. Previous studies have revealed that the CaMKII promoter predominantly drives transgene expression in excitatory glutamatergic neurons in the cortex, HPC, and amygdala [4,5,37,38]. In addition, brain regions such as the CA1, APC, LEnt, and Ment are strongly implicated in temporal lobe epilepsy (TLE) [39,40,41], rendering them more vulnerable to seizure induction relative to the VMH, PVN, and V1. Despite their established roles in TLE, the CEA [19] and LS [42] exhibited low seizure susceptibility, which may be linked to the high density of GABAergic inhibitory neurons in these regions. Meanwhile, BLA neurons send direct projections to GABAergic neuron-rich regions such as the striatum, BNST, and CeA, which may suppress seizure susceptibility [43,44,45]. These proposed mechanisms represent possible explanations for the observed regional heterogeneity in seizure susceptibility. Our findings alert researchers designing repeated in vivo optogenetic stimulation protocols that light pulses may trigger seizures or increase seizure vulnerability. Thus, optogenetic experiments must balance sufficient circuit activation against the risk of seizure generation.

Optogenetics is a powerful technology that enables optical control of cellular activity, and has been widely used to study neural circuits, brain diseases, and biomedical applications beyond the nervous system [1]. To date, more than 15,450 papers related to optogenetics have been published (based on PubMed data), and this number continues to grow. However, optogenetic simulation parameters vary considerably across studies, including those focused on epilepsy induction and treatment [7,8,9,11,12,13,14,29,33,46,47,48,49,50,51,52,53,54]. Our results emphasize the critical importance of rigorously controlling light-delivery parameters in optogenetic experiments.

## 5. Limitations of the Study

One limitation of the present study is that we exclusively employed a ChR2-based optogenetic approach. Although ChR2 is widely used, different opsins exhibit distinct kinetics and dynamic ranges, which may result in substantial differences in the optical parameters required to elicit seizures. Additionally, the use of AAV vectors driven by the CaMKII promoter represents another constraint; future studies should employ alternative promoters to further uncover potential side effects. Another limitation is that numerous behavioral paradigms remain to be tested to determine whether optogenetic stimulation generally influences animal task performance. Here, we provide only one example showing that ChR2-based activation of CaMKII^+^ neurons in the CA1 disrupts contextual fear memory. Moreover, other limitations of our study include the small and unequal group sizes, the absence of a formal a priori sample-size calculation, the exclusive use of male animals, the sequential non-randomized design of the parameter experiments, potential carry-over or kindling effects, limited spatial electrophysiological monitoring, and the inability to completely separate the effect of ADs from stimulation intensity in the fear-conditioning experiment. Further systematic studies are warranted to elaborate and extend these findings in greater detail.

## 6. Conclusions

In conclusion, while optogenetics is a powerful tool widely adopted in neuroscience and biomedical research, its potential to trigger seizure-like activity is heavily influenced by the heterogeneity of stimulation parameters across studies. Therefore, our findings highlight the critical need to rigorously control light-delivery protocols to ensure the validity and reproducibility of future in vivo optogenetic experiments.

## Figures and Tables

**Table 1:** Effects of the first trial of light stimulation on ChR2-expressing CaMKII^+^ neurons in different brain regions.

Target Brain Regions	LFP Recording Sites	Induced ADs/ Probability (%)	Behaviors in Response to Light	Seizure Behavior/ Racine Score
CA1	Right CA1, Bilateral DG, PC, BLA, EEG	Yes (90.9)	Immobility, freezing	Yes (1)
APC	Bilateral DG, PPC, EEG	Yes (80)	Freezing, head nodding	Yes (1, 2)
LEnt	Bilateral DG, PPC, EEG	Yes (85.7)	Immobility, freezing	Yes (1)
MEnt	Bilateral DG, PPC, EEG	Yes (83.3)	Immobility, freezing	Yes (1)
BLA	Bilateral DG, APC, EEG	No	No significant changes	No
CAE	Bilateral DG, APC, EEG	No	No significant changes	No
PVN	Bilateral DG, APC, EEG	No	Escape, flight	No
VMH	Bilateral DG, BLA, EEG	No	Escape, flight	No
LS	Bilateral DG, APC, EEG	No	No significant changes	No
V1	Bilateral DG, BLA, EEG	No	No significant changes	No

## Data Availability

Data are contained within the article.

## Supplementary Materials

The following supporting information can be downloaded at: https://www.mdpi.com/article/10.3390/biology15181669/s1, Figure S1: Brain-wide neuronal activation following single-trial optogenetic stimulation of the CA1 region; Figure S2: Optogenetic stimulation of CaMKII^+^ neurons lacking ChR2 expression in the CA1 fails to induce ADs; Figure S3: Repetitive optogenetic stimulation enhances seizure susceptibility in specific brain regions; Figure S4: Repetitive optogenetic stimulation of CaMKII^+^ neurons in the BLA enhances seizure susceptibility; Figure S5: Optogenetic activation of the APC–LEnt projection is sufficient to induce seizures; Figure S6: Long-term ChR2 Expression fails to alter seizure threshold induce by light stimulation.
