# Supplementary material for: Seizure-Inducible Risks of In Vivo Optogenetic Manipulations
Source: Biology (Basel). 2026 Sep 21;15(18):1669. doi: 10.3390/biology15181669 (PMC13604151; doi:10.3390/biology15181669)
Supplement: Supplementary file 1 [file biology-15-01669-s001.zip › Supplemental information titles and legends.pdf]

Supplemental information titles and legends

Figure S1. Brain-wide neuronal activation following single-trial optogenetic stimulation of the CA1 region

A-B: Representative immunofluorescent images showing c-Fos expression (green) after single-trial light stimulation of the CA1 region three weeks after AAV-CaMKII-ChR2-mCherry or AAV-CaMKII-mCherry injection. Nuclei were stained with DAPI. Scale bar: 200 μm.

Figure S2. Optogenetic stimulation of $CaMKII^+$ neurons lacking ChR2 expression in the CA1 fails to induce ADs

A: Schematic of viral injection, optical fiber / electrode implantation in freely moving mouse. AAV-CaMKII-mCherry was injected into the CA1 region. After three weeks, single-trial light stimulation (light power, 3 mW; frequency, 20 Hz; pulse width, 5 ms; duration, 10 s) was delivered simultaneously with LFP / EEG recording.

B. Representative images showing viral injection, light stimulation and recording sites. Nuclei were stained with DAPI. Scale bar: 200 μm.

C. Representative LFP / EEG raw traces (top) and time–frequency spectrograms (bottom). No light-evoked ADs were observed during or after light stimulation in the DG (left and middle panels) and cerebellum (right panel). Blue shading indicates light stimulation.

Figure S3. Repetitive optogenetic stimulation enhances seizure susceptibility in specific brain regions

A: Racine scores following four consecutive stimulations on days 1, 2 and 16 across four brain regions. n = 7 (CA1), n = 5 (APC), n = 8 (LEnt), n = 5 (Ment) mice.

B-C: Latency and duration of ADs following four consecutive stimulations on days 1, 2 and 16 across the four brain regions. Data are presented as mean ± SEM.

Figure S4. Repetitive optogenetic stimulation of CaMKII^{+} neurons in the BLA enhances seizure susceptibility

A: Schematic of the repetitive optogenetic experiment in the BLA. Virus was injected into the BLA. After three weeks, four consecutive optogenetic stimulations (Stim.1- Stim.4) were presented on day 1, and repeated on days 2 and 16. Light stimulation: power, 3 mW; frequency, 20 Hz; pulse width, 5 ms; duration, 10 s.

B: Representative LFP / EEG traces during and after single-trial light stimulation in the BLA. No ADs were detected on day 1. In contrast, minor and robust ADs were induced on days 2 and 16, respectively. Blue shading indicates light stimulation.

Figure S5. Optogenetic activation of the APC–LEnt projection is sufficient to induce seizures

A: Schematic of optogenetic stimulation experiment targeting the APC-LEnt projection. Virus was injected into unilateral APC and an optical fiber was implanted into the ipsilateral LEnt. After three weeks, single-trial light stimulation was delivered to the LEnt and LFPs were recorded from the ipsilateral DG. Light stimulation: power, 5 mW; frequency, 20 Hz; pulse width, 5 ms; duration, 15 s.

B: Representative histological images showing viral injection site in the APC and optical fiber position in the LEnt. Nuclei were stained with DAPI. Scale bar: 200 μm.

C: Representative LFP traces from the ipsilateral DG during and after single-trial optogenetic stimulation of the APC-to-LEnt projection. Typical ADs were recorded.

D: Schematic of the repetitive optogenetic simulation protocol targeting the APC-LEnt projection. Four consecutive stimulations (Stim.1–Stim.4) were delivered on day 1, and repeated on days 2-4. Light stimulation: power, 5 mW (optical fiber tip); frequency, 20 Hz; pulse width, 5 ms; duration, 15 s.

E: Mean Racine scores for the four consecutive daily stimulations on days 2 and 4 (n =2 mice). Data are presented as mean ± SEM.

Figure S6. Long-term ChR2 Expression fails to alter seizure threshold induce by light stimulation

A: Experimental schematic of viral injection, light stimulation, and EEG recording in freely moving mouse. AAV-CaMKII-ChR2-mCherry was injected into the CA1 region. After six weeks, light stimulations were delivered with gradually increased light power, duration, frequency and pulse width to determine the seizure threshold, following the same procedures as the three-week ChR2 expression experiments.

B: Light power required to induce the first AD following six-week ChR2 expression (n = 11 mice). Data are presented as mean ± SEM.

C: Probability of inducing ADs following incremental increases in light stimulation duration.

D: Probability of inducing ADs following incremental increases in light stimulation frequency.

E: Probability of inducing ADs following incremental increases in light stimulation pulse width.
